# Data-driven assessment of air quality and health benefits from future shipping emission controls in coastal China

**DOI:** 10.1016/j.eehl.2025.100203

**Published:** 2025-11-26

**Authors:** Zhenyu Luo, Zhaofeng Lv, Tingkun He, Wen Yi, Yongyue Wang, Kebin He, Huan Liu

**Affiliations:** State Key Laboratory of Regional Environment and Sustainability, School of Environment, Tsinghua University, Beijing 100084, China

**Keywords:** Shipping emission, SECA, NECA, Air quality, Health burden

## Abstract

The lack of toolkits for assessing the shipping-related atmospheric impacts limits China's ability to formulate effective shipping emissions control policies to address coastal air pollution and mitigate related health burdens. Here, we developed a deep learning model, DeepShip, to efficiently predict shipping-related PM_2.5_ concentrations and further coupled it with a multi-task learning and generative-adversarial training strategy to enhance the sensitivity of the data-driven model to variations in small emission sources. Based on DeepShip, we comprehensively analyzed the response of shipping-related PM_2.5_ to changes in anthropogenic emissions based on 210 scenarios involving emission reductions of shipping and land-based sectors. Furthermore, sulfur and nitrogen emission control scenarios that China might implement in the future were established to assess their cost, air quality improvement, and health benefits. We found that shipping-related PM_2.5_ shows an almost linear relationship with shipping emissions, while exhibiting a nonlinear relationship with land-based emissions. Considering the cost and environmental-health benefits, future shipping emissions control should prioritize progressively enhancing the NO_*x*_ emission standard while coordinating with land-based emission reductions.

## Highlights

• A deep learning model was developed to predict shipping-related PM_2.5_ pollution.
• 210 scenarios were established to investigate the source-receptor relationship.
• Shipping NO_x_ emission control would offer considerable environmental-health benefits.

## Graphical abstract

**Figure:**
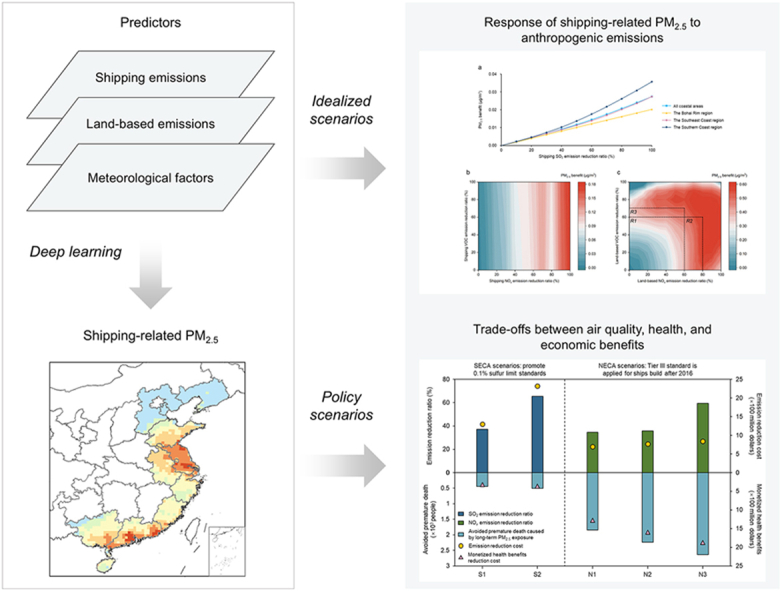

## 1 Introduction

Shipping emissions could worsen air pollution and threaten public health [[1], [2], [3]]. Although China's progressively promoted Chinese Shipping Sulfur Emission Control Area (SECA) policy from 2016 to 2019 reduced shipping SO_2_ emissions by 29.6% [4], the focus on a single pollutant and the limited reduction amount have prevented it from coordinating with land-based anthropogenic emission reduction processes [5]. Furthermore, with the trends of population migration towards the coast and population aging in China, even though the global sulfur cap that took effect in 2020 further reduced SO_2_ emissions by 70%, the shipping-related premature deaths attributed to PM_2.5_ long-term exposure in 2020 still increased by 11.4% compared with the level associated with uncontrolled shipping emissions in 2016 [6]. However, apart from promoting marine fuel with a sulfur content not exceeding 0.1% m/m (hereinafter referred to as ultra-low sulfur fuel) in Hainan in 2022, China has not announced additional shipping emission control policies. In the context of increasing maritime trade, continuous changes in population structure, and further reductions in land-based anthropogenic emissions, such as the nationwide implementation of the China VI standard [7], the relative contribution of shipping emissions to both air quality and public health would further increase. Therefore, China still faces significant pressure to reduce shipping emissions.

Shipping emission control guided by environmental health goals requires sufficient scientific evidence to support it, as a decrease in emissions does not necessarily translate into improved air quality. A typical example is the COVID-19 pandemic, during which transportation activities were nearly halted and the associated NO_*x*_ emissions were significantly reduced. However, severe pollution events still occurred in the North China region due to unbalanced emission reductions between transportation and industrial sectors [8]. Therefore, policy-making needs to fully consider the response of concentrations to emissions. However, only a limited number of studies [9,10] have explored the air quality and health benefits of China's future control policies targeting shipping sulfur and nitrogen emissions. Although several international studies [[11], [12], [13]] have provided insights and experiences related to the Nitrogen Emission Control Area (NECA) and SECA, these studies generally focus on limited policy scenarios and do not consider changes in emissions from other sectors. The lack of comprehensive scenario simulations of the nonlinear response of concentrations to changes in both land-based and shipping emissions greatly limits the formulation of shipping emission control policies in the context of multi-sectoral emission reduction coordination.

Although comprehensive scenario simulation is theoretically possible, it is extremely challenging to implement due to the computational intensity of Chemical Transport Models (CTMs). With advances in data science [14], mathematical models involving machine learning [15] and deep learning [16] are increasingly replacing traditional physical models for predicting air pollutant concentrations and analyzing their nonlinear responses to emissions. Existing models typically simulate the total air pollutant concentrations in response to changes in anthropogenic emissions from all sectors. However, due to a lack of prior knowledge on atmospheric physical and chemical processes, data-driven models may struggle to capture real-world patterns and often suffer from limited accuracy [17]. This is particularly significant for shipping, a relatively small contributor among anthropogenic emissions with national shares of SO_2_ and NO_*x*_ of about 13% in 2017 [18]. The concentration caused by its emissions may even fall within the margin of bias in such models. Therefore, simulating shipping-related pollutant concentrations and their response to changes in shipping emissions imposes higher demands on the accuracy and sensitivity of data-driven models.

In summary, developing an air quality simulation system that balances high efficiency and accuracy is essential for investigating the source-receptor relationships between shipping emissions and air quality, and further identifying environmentally oriented pathways for shipping emissions control. Therefore, we developed a deep learning model (DeepShip) to predict shipping-related PM_2.5_ concentrations, using multi-year CTM simulation data from our previous study [6]. To capture the concentration variations driven by changes in small emission sources such as the shipping sector, multi-task learning and generative-adversarial training strategies were incorporated to enhance the model's accuracy. Based on DeepShip, we comprehensively assessed the source-receptor relationships between anthropogenic emissions and shipping-related PM_2.5_. Furthermore, we evaluated the cost-effectiveness of potential SECA and NECA scenarios that China might implement based on current emission levels. This research deepens the understanding of the source-receptor relationship between anthropogenic emissions and shipping-related air pollution and provides new insights into coordinated control of anthropogenic emissions.

## 2 Materials and methods

### 2.1 Framework for DeepShip

**Fig. 1:**
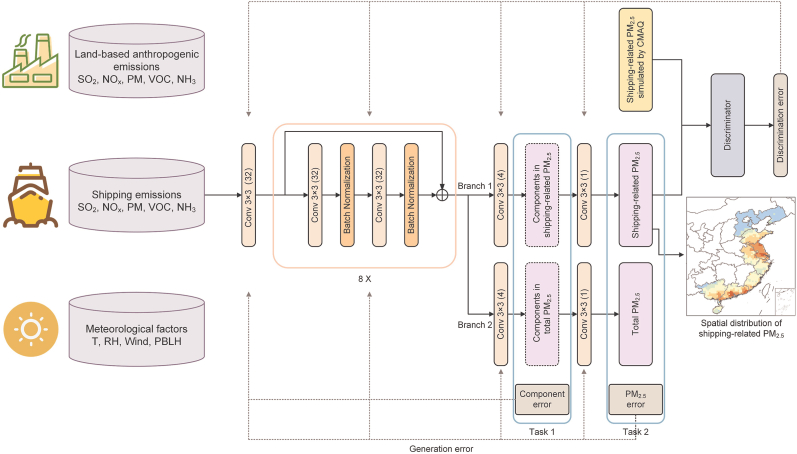
The framework of DeepShip.

Deep neural networks perform well in approximating complex nonlinear functions. Moreover, convolutional neural networks (CNNs) can effectively integrate the spatial relationships among different predictive indicators in adjacent grids, allowing for the capture of local pollutant concentrations [16,19,20]. Therefore, this study developed a deep CNN-based model, DeepShip (Fig. 1), with residual blocks that provide the high precision needed for simulating complex atmospheric chemical processes. DeepShip includes two parts: a generator (G) and a discriminator (D). The task of G is to predict shipping-related PM_2.5_ based on the predictors (F). F includes land-based anthropogenic emissions (land-based mobiles, industry, power, domestic, and agriculture emissions; SO_2_, NO_*x*_, VOC, PM, and NH_3_), shipping emissions (SO_2_, NO_*x*_, VOC, and PM), and meteorological factors (10-m wind speed, 10-m wind direction, 2-m temperature, relative humidity, and planetary boundary layer height). Here, all data were directly obtained from the Community Multiscale Air Quality (CMAQ) simulation from our previous study [6], including emissions, pollutant concentrations, and meteorological variables. Although the meteorological data are originally generated by Weather Research and Forecasting (WRF), they remain in the output files of the CMAQ. These data were directly used for building the DeepShip model without any additional preprocessing. Details are provided in the next section.

One of the key components of DeepShip is the discriminator (D), which, together with the generator (G), forms a Generative Adversarial Network (GAN). GAN is a classical generative model in deep learning, composed of the adversarial relationship between G and D, where their interaction enhances overall model performance [21]. In DeepShip, G predicts the shipping-related PM_2.5_, while D distinguishes between the shipping-related PM_2.5_ generated by G and simulated by CMAQ (truth value). The D functions as a binary classifier, with its structure illustrated in Fig. S1 and a detailed description in the Supplementary Material Discriminator Model Description.

Multi-task learning is a training strategy designed to enhance model performance by simultaneously learning multiple related tasks. In this study, DeepShip incorporates two tasks: predicting PM_2.5_ component concentrations (Task 1) and predicting PM_2.5_ concentrations (Task 2). As shown in Fig. 1, after the eight residual modules in the generator (G), there is a model branch (Branch 2) that is identical to the network structure of Branch 1. Branch 1 outputs the shipping-related PM_2.5_ and its components, while Branch 2 outputs the total PM_2.5_ and its components. Here, the components include sulfate, nitrate, secondary organic matter, and primary organic matter. The goal of Task 1 is to minimize the training error for predicting component concentrations of both total and shipping-related PM_2.5_, and the goal of Task 2 is to minimize the training error for predicting both total and shipping-related PM_2.5_.

In this study, we trained four independent models based on simulation data for January, April, July, and November. The data from 2016 to 2019 were used for training, while the most extreme samples in terms of emission and concentration changes from 2020 were used to evaluate the models' performance. To enhance training efficiency and accuracy, we divided the training process into two stages: pre-training and formal training. In the pre-training stage, only the generator G was trained. The AdamOptimizer algorithm was employed for training. During this phase, the learning rate was set to 0.0001, and the maximum number of iterations was set to 100. In the formal training stage, both the generator G and the discriminator D were trained simultaneously. The AdamOptimizer algorithm was also employed in this phase. Here, the learning rate decays with the increase in the number of iterations (iter), set as 0.0001 ​× ​0.99^iter^, while the maximum number of iterations was set to 2000. When the model performance stabilized as the number of iterations increased, it was deemed that the model converged, and training was halted.

### 2.2 Configuration of CMAQ model

The Weather Research and Forecasting (WRF, version 3.8.1)−Community Multiscale Air Quality (CMAQ, version 5.4) model was applied to simulate the air quality in China during January, April, July, and November from 2016 to 2020, which represented winter, spring, summer, and fall, respectively. The modeling domain covered all of China and some parts of East Asia with a horizontal resolution of 36 ​km ​× ​36 ​km, including all highly developed port city clusters of China.

Atmospheric gas-phase chemistry in the CMAQ was simulated with the SAPRC07tic chemical mechanism, and aerosols were predicted using the AERO7. The chemical boundary conditions of CMAQ inputs were collected from the Community Atmosphere Model with Chemistry (CAM-chem) simulation output of global tropospheric and stratospheric compositions [22]. The Integrated Source Apportionment Method (ISAM) was applied to determine the source contribution to the ambient PM_2.5_ and its species concentrations. We divided the emissions into five groups to trace them separately in the ISAM: the land-based anthropogenic emissions (emissions from the mobile, industrial, power, domestic, agricultural, and open-burning sources), the river vessels (RVs) emissions, the coastal vessels (CVs) emissions, the ocean-going vessels (OGVs) emissions, and other emissions (natural source emissions and the anthropogenic emissions from other countries within the modeling domain). The details of emissions used in the CMAQ model are shown in Table S1.

For CMAQ-related data used in DeepShip, the shipping-related PM_2.5_ was calculated as the sum of contributions from RVs, CVs, and OGVs to PM_2.5_, while the total PM_2.5_ was calculated as the sum of contributions from the five major emission sectors together with the PM_2.5_ of initial and boundary conditions. It should be noted that the PM_2.5_ mentioned in this study includes both primary and secondary components. The shipping emissions are defined as the sum of RVs, CVs, and OGVs emissions. Although shipping and land-based anthropogenic emissions are available at relatively high spatial resolution, in this study, all emissions were regridded to the WRF-CMAQ resolution of 36 ​km to ensure consistency. Therefore, the dimension of the output of DeepShip (shipping-related PM_2.5_) was 136 ​× ​137, while the input dimensions were 136 ​× ​137 ​× ​5 for land-based anthropogenic emissions, 136 ​× ​137 ​× ​4 for shipping emissions, and 136 ​× ​137 ​× ​5 for meteorological variables.

### 2.3 Random forest model

Here, the Random Forest (RF) model was used to predict shipping-related PM_2.5_. The input predictor variables included land-based anthropogenic emissions (SO_2_, NO_*x*_, VOC, PM, and NH_3_), shipping emissions (SO_2_, NO_*x*_, VOC, and PM), and meteorological factors (10-m wind speed, 10-m wind direction, 2-m temperature, relative humidity, and planetary boundary layer height). For each sample, the values of land-based anthropogenic and shipping emissions were calculated as the sum of emissions from the three neighboring CMAQ grid cells.

In the RF model, the number of predictors randomly sampled at each split node in the decision tree and the number of trees to grow are two important hyperparameters that determine the performance of the model. By comparing the mean squared error for test datasets across models with candidate parameter combinations, we set them to 10 and 200 in the RF model, respectively. Additionally, a 10-fold cross-validation repeated 10 times was used to evaluate the prediction performance of our models.

### 2.4 Scenario setting

We used the 2020 levels of shipping and land-based anthropogenic emissions as baseline emissions and established 210 idealized emission-reduction scenarios to quantify the response of shipping-related PM_2.5_ to changes in anthropogenic emissions, as outlined in Table S2. Among them, S1 to S10 represent shipping SO_2_ reduction scenarios, corresponding to reductions of 10%–100% in shipping SO_2_ emissions, aimed at examining the relationship between the shipping-related PM_2.5_ and shipping SO_2_ emissions. Due to the mutual changes in NO_*x*_ and VOC emissions affecting atmospheric chemical properties, and subsequently influencing the formation of secondary particulate matter, we considered the synergistic effects of shipping NO_*x*_ and VOC emissions reductions, establishing scenarios N1V1 to N10V10, which represent reductions of 10%–100% in both shipping NO_*x*_ and VOC emissions. Given that land-based anthropogenic emissions have more significant impacts on atmospheric chemical properties, we also examined the effects of synergistic reductions in land-based anthropogenic NO_*x*_ and VOC emissions on shipping-related PM_2.5_, establishing scenarios LN1V1 to LN10V10, which represent reductions of 10%–100% in these emissions. In these scenarios, the emission reductions were idealized. For the shipping reduction scenarios, emissions were uniformly reduced at all grid cells within 200 Nm from the territorial sea baseline of the Chinese mainland. For the land-based anthropogenic reduction scenarios, emissions were uniformly reduced at all grid cells across mainland China.

Starting from January 1, 2022, according to China's newly issued shipping emission control policies, ships operating in the coastal control areas of Hainan must use marine fuel with a sulfur content not exceeding 0.1% m/m. However, this has not yet been implemented at the national level. For NO_*x*_ emissions, China has not yet established an NECA, but, as found in previous studies, shipping-related PM_2.5_ has shifted toward nitrate-dominated pollution, indicating a need for future shipping NO_*x*_ emission control [6]. Therefore, based on current shipping emission control standards and pollution conditions, we used the 2020 levels of shipping and land-based anthropogenic emissions as the baseline scenario and established five potential policy scenarios to be implemented in the future, as shown in Table S3. Scenario S1 requires ships operating under any conditions within 12 Nm of the Chinese territorial baseline to use ultra-low sulfur fuel, while scenario S2 extends the requirement to 200 Nm. Scenarios N1, N2, and N3 are NECA control policies that mandate new ships built after 2016 to comply with stricter Tier III emission standards, with N1 applicable to all port areas in China, N2 to waters within 12 Nm of the territorial baseline, and N3 to waters within 200 Nm. It should be noted that in these idealized emission-reduction scenarios, the reduction rule assumes emissions are uniformly reduced by the same proportion in each grid.

Shipping emission inventories (0.05° ​× ​0.05°, monthly) around China were built based on global automatic identification system data and the updated Shipping Emission Inventory Model (SEIM v.2.0) [4]. In SEIM v.2.0, shipping emissions for air pollutants (for example, SO_2_, PM, NO_*x*_, CO, and HC) from the main engines, auxiliary engines, and boilers were calculated. A detailed description of SEIM is provided in the Supplementary Material Shipping Emission Inventory Model.

### 2.5 Emission-reduction cost

Currently, widely used technologies for shipping NO_*x*_ emission reduction primarily include fuel optimization, combustion pretreatment, and exhaust gas aftertreatment. However, existing literature shows significant variability in the emission-reduction ratios of different technologies surveyed, and there is currently no comprehensive assessment model to estimate the economic costs. In this context, we summarized the relationship between NO_*x*_ reduction costs and reduction ratios from previous studies [[23], [24], [25]] (Table S4), and applied nonlinear fitting to predict the NO_*x*_ reduction costs for scenarios N3, N4, and N5, as shown in Fig. S2.

When transitioning from Tier II to Tier III standards, the NO_*x*_ emission factor for ships decreases by approximately 80% [26]. Thus, the implementation of NECA policies is assumed to achieve an 80% NO_*x*_ reduction ratio per ship. Substituting this reduction ratio into the relationship expression in Fig. S2 yields a NO_*x*_ reduction cost of USD 47 per kW. Then, we estimated the total power of ships built after 2016 within the scope of all ports in China, within 12 Nm and 200 Nm of the territorial baseline (Table S5), according to the NECA implementation range and ship data from scenarios N3, N4, and N5, and calculated the total reduction costs for different scenarios.

Generally, the costs associated with switching fuels for ships include fuel price premiums, vessel facility retrofit costs, and operational costs. Compared with the fuel price premiums, facility retrofit and operational costs are significantly lower. Therefore, given data availability, we accounted only for fuel price premiums and estimated the reduction costs for scenarios S1 and S2, using the fuel consumption method.

$$R=\sum P_{\text{actual}}\times {\text{SFOC}}_{\text{load}}\times \Delta t$$

Cost=R×Price

where, *R* represents the fuel consumption, $P_{\text{actual}}$ (kWh) and ${\text{SFOC}}_{\text{load}}$ (g/kWh) represent the actual power of the ship and adjusted SFOC (specific fuel oil consumption) based on the ship's engine load, respectively, Δt (h) is the duration for the engine, *Cost* is the emissions-reduction cost, *Price* (USD/ton) is the fuel price premiums.

In this study, the annual fuel consumption for ships within 12 and 200 Nm of the territorial baseline of China was estimated to be 9.5 ​Mt and 16.9 ​Mt, respectively. Since no ultra-low sulfur fuels were available for sale in the East Asia region, the average fuel prices at the Port of Singapore from October 2023 to April 2024 were used as a reference. The average prices for low-sulfur fuel (sulfur content not exceeding 0.5% m/m) and ultra-low-sulfur fuel were USD 643 per ton and USD 800 per ton, respectively (source: https://shipandbunker.com/prices/apac/sea/sg-sin-singapore). Another report indicated that the price difference between the two types of fuel ranged from USD 70 per ton to USD 200 per ton. Therefore, this study set the ultra-low sulfur fuel premium at USD 135 per ton, based on the average fuel price premium as a reference.

### 2.6 Health benefit

Here, we quantified the health benefits by assessing the premature mortality due to long-term exposure to PM_2.5_ between each scenario and the baseline. The health impacts of long-term exposure to PM_2.5_ were estimated based on population data and cause-specific cross-sectional mortality rates with a detailed age structure. Four health endpoints related to long-term PM_2.5_ exposure were covered, including premature mortality due to chronic exposure caused by chronic obstructive pulmonary disease, ischemic heart disease, stroke, and lung cancer. Details are provided in the Supplementary Material, Mortality estimation [6].

The willingness-to-pay (WTP) method is an economic approach used to assess individuals' or households' willingness to pay for non-market goods or services. It is widely applied in environmental economics to estimate the value of improvements in air quality, reduced disease risk, and enhanced ecosystems. Here, we employed the WTP method to evaluate the economic losses caused by PM_2.5_ pollution attributed to shipping emissions, representing the loss of environmental value. The Value of Statistical Life (VSL) is a commonly used parameter in assessing economic losses, indicating how much individuals are willing to pay to reduce the risk of death. This study utilized the VSL data for China in 2020 from Xie et al. [27] to evaluate the economic losses caused by shipping-related PM_2.5_ pollution.

### 2.7 Limitations and uncertainties

This study recognized several key limitations and uncertainties in both the modeling framework and the results:

1) The training data for the DeepShip model relied on simulations from a CTM, which may introduce systematic biases due to assumptions about meteorological conditions, emission inventories, and chemical reaction mechanisms in the CTM. In addition, not all potential contributors to PM_2.5_, such as natural source emissions, were considered in DeepShip. Instead, the model focuses on learning the dominant relationships embedded in the available training data. While some minor contributions may be omitted, the overall conclusions regarding shipping-related PM_2.5_ remain robust.
2) DeepShip was trained and validated using simulated data from 2016 to 2020. During this period, global fuel sulfur content regulations significantly reduced shipping-related SO_2_ emissions, despite increases in shipping NO_*x*_ and VOC emissions. Although the neural network can respond to conditions beyond the range of its training data, DeepShip may introduce bias when evaluating scenarios for shipping NO_*x*_ and VOC emission reductions. Similarly, reductions in land-based anthropogenic emissions during this period were limited, which may also affect DeepShip's performance when evaluating scenarios for land-based anthropogenic NO_*x*_ and VOC emission reductions.
3) The emission reduction scenarios assume uniform reductions across all grid cells, which may result in uncertainties in source-receptor relationships.
4) The health impact assessment focuses on long-term exposure to PM_2.5_ and its association with four major diseases, while omitting short-term health risks and the co-effects of other pollutants such as SO_2_ and ozone. Future work should incorporate these acute effects and multi-pollutant interactions to enable a more comprehensive and persuasive policy analysis.
5) Future changes in population size, spatial distribution, and demographic structure are not accounted for in the model, which may bias estimates of the long-term public health benefits of emissions policies.

## 3 Results and discussion

### 3.1 Performance of DeepShip

Here, we compared the performance of DeepShip with its two variants (DeepShip-1 and DeepShip-2) and the RF model, in predicting shipping-related PM_2.5_. DeepShip-1 is a simplified version of DeepShip, keeping only the multi-task learning mechanism but without the GAN. DeepShip-2 further simplifies DeepShip-1, being a single-task model that only includes Task 2. The RF model used the same data samples and predictors as DeepShip; details of the RF model are presented in the Section 2.3.

**Fig. 2:**
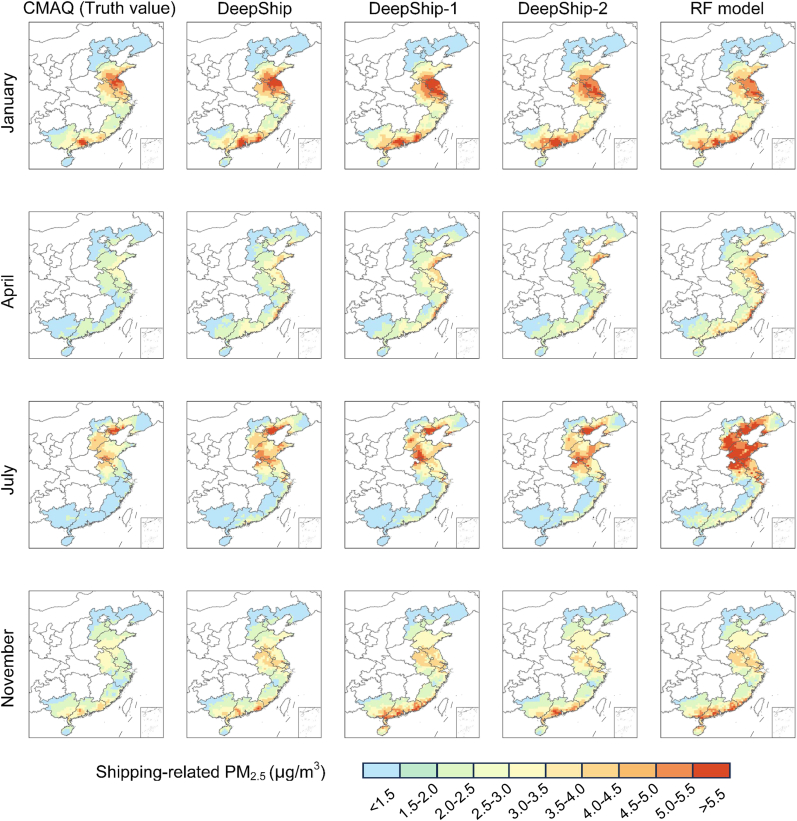
The shipping-related PM_2.5_ from CMAQ, DeepShip, DeepShip-1, DeepShip-2, and the RF model.

Fig. 2 and Table 1 show the monthly average shipping-related PM_2.5_ and the performance metrics of different models, respectively. Overall, DeepShip, DeepShip-1, and DeepShip-2 improved the dispersal of high-value areas seen in the RF model, particularly in the simulations for the Bohai Rim and Shandong Peninsula regions in January and July. For example, in July, DeepShip-2 achieved MB, MAE, and RMSE values of 0.53 μg/m^3^, 0.57 μg/m^3^, and 0.84 μg/m^3^, respectively, which are significantly better than the corresponding values of 1.36 μg/m^3^, 1.36 μg/m^3^, and 1.70 μg/m^3^ from the RF model. With the introduction of the multi-task learning strategy, DeepShip-1 performed better on several metrics than DeepShip-2, demonstrating that multi-task learning can improve prediction accuracy by incorporating related tasks. However, in April, DeepShip-1 exhibited relatively larger simulation errors. DeepShip systematically reduced the overestimation observed in DeepShip-1 and DeepShip-2, which is especially evident in the SC region in November. Even in April, DeepShip outperformed DeepShip-2, which itself outperformed DeepShip-1, across all error metrics, confirming that the generative adversarial mechanism contributes to improved accuracy and generalization. These results indicate that the training strategies can enhance the model's accuracy, allowing it to more closely approximate smaller concentration values by adding strong constraints.

**Table 1:** Performance of different models.

Time	Model	MB	MAE	MSE	RMSE	RE	NME	NMGE	MFB	MFE
Jan.	DeepShip	0.22	0.37	0.32	0.57	0.23	0.10	0.17	0.07	0.21
	DeepShip-1	0.44	0.50	0.48	0.69	0.46	0.20	0.23	0.24	0.28
	DeepShip-2	0.52	0.54	0.51	0.72	0.34	0.24	0.25	0.18	0.29
	RF	0.46	0.53	0.56	0.75	0.37	0.21	0.25	0.24	0.27
Apr.	DeepShip	0.40	0.43	0.34	0.58	0.29	0.26	0.28	0.22	0.24
	DeepShip-1	0.50	0.52	0.49	0.70	0.32	0.33	0.33	0.25	0.26
	DeepShip-2	0.43	0.46	0.43	0.66	0.32	0.28	0.30	0.23	0.25
	RF	0.86	0.87	1.14	1.07	0.58	0.56	0.56	0.42	0.42
Jul.	DeepShip	0.38	0.47	0.51	0.71	0.39	0.22	0.28	0.21	0.28
	DeepShip-1	0.39	0.50	0.59	0.77	0.43	0.23	0.29	0.21	0.29
	DeepShip-2	0.53	0.57	0.71	0.84	0.48	0.31	0.33	0.29	0.31
	RF	1.36	1.36	2.89	1.70	1.17	0.79	0.80	0.61	0.61
Nov.	DeepShip	0.31	0.36	0.27	0.52	0.20	0.17	0.19	0.13	0.17
	DeepShip-1	0.32	0.37	0.30	0.55	0.22	0.17	0.20	0.15	0.18
	DeepShip-2	0.47	0.48	0.37	0.61	0.32	0.25	0.25	0.24	0.25
	RF	0.60	0.61	0.73	0.85	0.33	0.32	0.33	0.25	0.26

MB, Mean bias; MAE, Mean absolute error; MSE, Mean square error; RMSE, Root mean square error; RE, Relative error; NME, Normalized mean error; NMGE, Normalized mean gross error; MFB, mean fractional bias; MFE, mean fractional error.

Additionally, DeepShip and CMAQ simulations exhibit a high correlation, with an average R^2^ exceeding 0.85 (Fig. S3), while DeepShip also greatly reduces the computational demand, from the day-scale required by CMAQ to the minute-scale on a standard desktop for a one-month simulation, showing its potential for large-scale scenario assessments. Specifically, for the 210 idealized emission reduction scenarios considered in this study (a total of 210 ​× ​4 ​= ​840 months), CMAQ-ISAM would require approximately 29,160 ​h on 56 cores. It should be noted that this estimate also includes time for data input/output operations within CMAQ-ISAM, which cannot be separately evaluated. By contrast, applying DeepShip required only about 42 ​h in total on a desktop equipped with an NVIDIA GeForce RTX 2060 SUPER GPU (8 ​GB memory), demonstrating the “train once, apply many times” advantage of DeepShip.

### 3.2 Response of shipping-related PM_2.5_ to changes in anthropogenic emissions

**Fig. 3:**
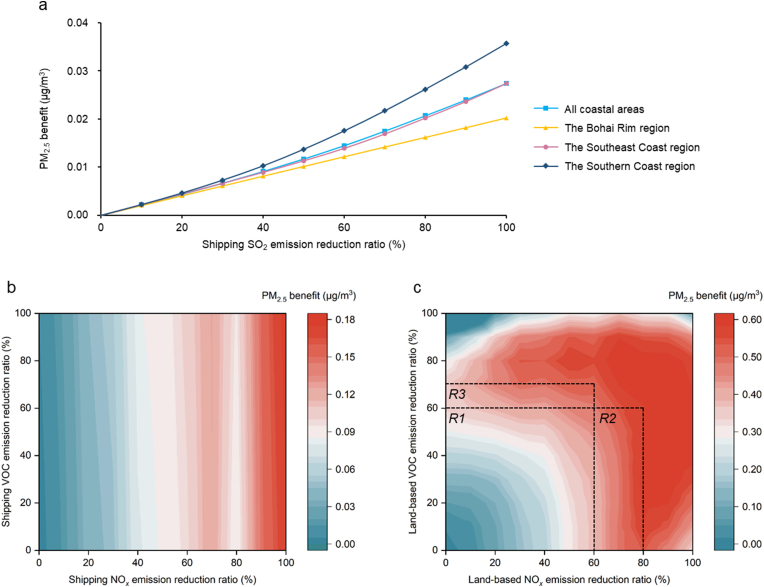
The response of shipping-related PM_2.5_ benefit to changes in (a) shipping SO_2_ emission, (b) shipping NO_*x*_ and VOC emissions, and (c) land-based NO_*x*_ and VOC emissions.

Here, we used the trained DeepShip to conduct 210 sets of idealized emission-reduction scenarios listed in Table S2 to investigate the response of shipping-related PM_2.5_ to changes in anthropogenic emissions. As shown in Fig. 3, the response relationship was defined as the relationship between the PM_2.5_ benefits and the corresponding reduction ratios, based on these idealized scenarios. The PM_2.5_ benefits were calculated as the difference in annual mean PM_2.5_ concentrations (averaged over January, April, July, and November) between the baseline scenario and idealized scenarios (BASE–idealized). The baseline simulation was conducted using anthropogenic emissions for the year 2020.

Fig. 3a shows the shipping-related PM_2.5_ benefits across all coastal areas in China (including all coastal provinces and municipalities), the Circum-Bohai Sea region (CBS, Liaoning, Hebei, Shandong, and Tianjin), the Southeast Coast region (SEC, Jiangsu, Zhejiang, Fujian, and Shanghai), and the Southern Coast region (SC, Guangdong, Guangxi, and Hainan) under different scenarios of shipping SO_2_ emission reductions. As shipping SO_2_ emissions gradually decrease, the shipping-related PM_2.5_ exhibits an almost linear downward trend. However, as previous studies have indicated, the concentration of sulfate in shipping-related PM_2.5_ has already dropped to less than 1.00 ​μg/m^3^. Therefore, for all coastal areas, even a 100% reduction in shipping SO_2_ emissions would only reduce the shipping-related PM_2.5_ by approximately 0.03 ​μg/m^3^. These PM_2.5_ benefits can vary significantly across different coastal regions. In the SC region, emissions from ocean-going vessels are relatively high, and sulfate (SO_4_^2−^) contributes to a large proportion of shipping-related PM_2.5_ [6]. Therefore, reducing shipping SO_2_ emissions leads to more significant improvements in air quality, with a maximum decrease in shipping-related PM_2.5_ of 0.04 ​μg/m^3^ observed under a 100% reduction in shipping SO_2_ emissions. In contrast, in the CBS region, where sulfate constitutes a smaller share of shipping-related PM_2.5_, the maximum concentration benefit is around 0.02 ​μg/m^3^.

Fig. 3b shows the contour plots of annual average shipping-related PM_2.5_ benefits in response to reductions in shipping NO_*x*_ and VOC emissions (CBS, SEC, and SC as shown in Fig. S4). Compared to VOCs, shipping-related PM_2.5_ is more sensitive to changes in shipping NO_*x*_ emissions and decreases almost linearly as shipping NO_*x*_ emissions are reduced. Given the high proportion of nitrate in shipping-related PM_2.5_, the air quality benefits of reducing shipping NO_*x*_ emissions across all coastal areas are more significant than those for SO_2_, with reductions reaching up to 0.18 ​μg/m^3^ when achieving a 100% reduction in shipping NO_*x*_ emissions. It is noteworthy that when the proportion of shipping NO_*x*_ emissions reductions reaches around 80%, there is a slight decrease in shipping-related PM_2.5_ benefits before they continue to increase. This may be due to the temporary increase in atmospheric oxidative capacity caused by unbalanced reductions in NO_*x*_ and VOC emissions from shipping sources themselves, or in comparison with land-based sectors whose emissions remain unchanged. Therefore, the increased atmospheric oxidative capacity promotes the formation of secondary components in PM_2.5_. This phenomenon is most evident in the CBS region and least evident in the SC region (Fig. S4). The stronger impact in the CBS region is due to the higher land-based anthropogenic emissions there, making the effects of unbalanced changes in shipping and land-based emissions on atmospheric chemistry more significant.

Although secondary organic matter (SOM) also constitutes a significant portion of shipping-related PM_2.5_, changes in shipping VOC emissions have minimal impact on concentration reduction across all coastal areas. Overall, under any level of shipping NO_*x*_ reduction, the air quality benefits of reducing shipping VOC emissions do not exceed 0.03 ​μg/m^3^. This is because the formation of SOM in PM_2.5_ is more dependent on precursors from land-based anthropogenic sources, such as road transportation and industrial emissions [28]. These precursors react with oxidants in the atmosphere to form secondary organic aerosols. Even with significant reductions in shipping VOC emissions, the lack of reductions in land-based anthropogenic emissions means that sufficient precursors remain available to promote SOM formation.

Due to the higher emissions, changes in land-based emissions have a more pronounced impact on atmospheric oxidative capacity. Therefore, shipping-related PM_2.5_ is more sensitive to changes in land-based NO_*x*_ and VOC emissions. For all coastal areas, the coordinated reduction of land-based NO_*x*_ and VOC emissions can reduce shipping-related PM_2.5_ concentrations by up to 0.64 ​μg/m^3^. Specifically, the maximum concentration benefit from independent reductions in land-based NO_*x*_ emissions is 0.54 ​μg/m^3^, while for VOCs, it is 0.36 ​μg/m^3^. Different from the linear response observed in shipping emission-reduction scenarios, there is a clear nonlinear relationship between shipping-related PM_2.5_ and changes in land-based NO_*x*_ and VOC emissions, with significant regional differences (Figs. 3c and S5). Across all coastal areas, shipping-related PM_2.5_ benefit generally increases as land-based NO_*x*_ and VOC emissions are reduced. Specifically, when both reduction ratios of NO_*x*_ and VOC emissions are below 60% (R1), the formation of ozone and hydroxyl radicals is jointly regulated by NO_*x*_ and VOCs. Under these conditions, the PM_2.5_ formation is sensitive to both precursors, and the PM_2.5_ benefits increase significantly with simultaneous reductions in NO_*x*_ and VOC emissions. When NO_*x*_ reductions exceed 60% (R2), PM_2.5_ becomes sensitive mainly to NO_*x*_ reductions, and further reductions generally decrease nitrate formation. However, once NO_*x*_ reductions approach 80%, such benefits begin to decline slightly, as the weakening of NO titration and radical loss may cause temporary increases in ozone and OH, thereby enhancing the conversion of SO_2_ to sulfates and VOCs to SOM. Similarly, when VOC reductions exceed 60% (R3), atmospheric chemistry shifts toward a NO_*x*_ -limited regime, and PM_2.5_ becomes mainly sensitive to VOC reductions, but the benefits decrease once VOC reductions exceed 70%.

For different coastal regions, the nonlinear relationship between shipping-related PM_2.5_ and land-based emissions in the SEC regions is similar to that of all coastal areas. In the CBS region, simultaneous reductions of shipping NO_*x*_ and VOC emissions yield greater improvements in shipping-related PM_2.5_ than reductions in either pollutant alone. However, in the SC region, this non-linear relationship is more complex: When land-based anthropogenic NO_*x*_ emissions reductions are less than 40%, shipping-related PM_2.5_ first decreases and then increases with VOC emissions reductions, although the maximum concentration benefit remains very limited. When NO_*x*_ emissions reductions exceed 40%, shipping-related PM_2.5_ increases as VOC emissions decrease. By contrast, the shipping-related PM_2.5_ consistently decreases with NO_*x*_ emission reductions when the reduction level is below 90%.

### 3.3 Cost-benefits of potential shipping emission control scenarios

We established potential shipping emission control scenarios that could be implemented in China, including SECA scenarios that require a 0.1% sulfur limit standard within 12 Nm (S1) and 200 Nm (S2) of the Chinese coastline, and NECA scenarios that apply the Tier III standard within port areas (N1), 12 Nm (N2), and 200 Nm (N3) of the Chinese coastline for ships built after 2016. We analyzed the emission-reduction potential, public health benefits, and monetized cost-effectiveness of these scenarios.

**Fig. 4:**
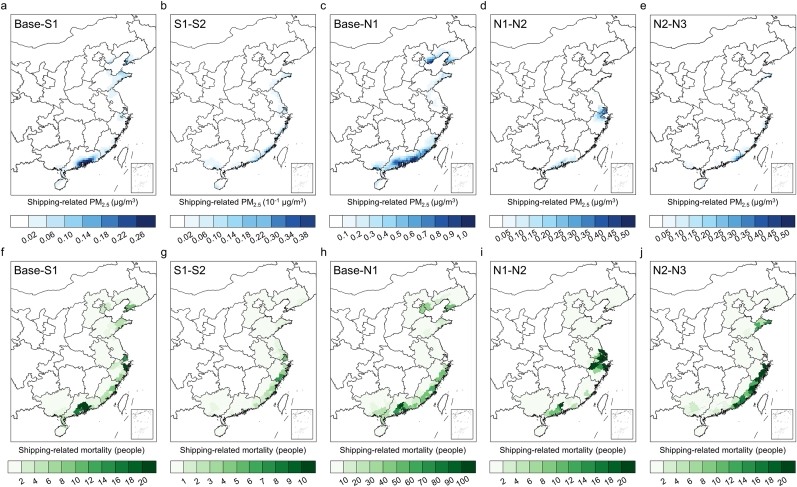
Changes in (a–e) shipping-related PM_2.5_ and (f–j) mortality between (a, f) Base and S1 scenarios, (b, g) S1 and S2 scenarios, (c, h) Base and N1 scenarios, (d, i) N1 and N2 scenarios, and (e, j) N2 and N3 scenarios.

Although the global fuel switch in 2020 significantly reduced shipping SO_2_ emissions, scenarios S1 and S2 can still achieve considerable SO_2_ emission reduction benefits, with reductions of 37% and 65%, respectively. The spatial characteristics of emission changes are shown in Fig. S6. However, the average shipping-related PM_2.5_ concentration of 2.25 ​μg/m^3^ across all coastal areas decreases by only 0.02 ​μg/m^3^ compared to the BASE scenario, with no significant improvement compared to the S1 scenario, except for some coastal cities in Fujian Province in the SEC region (Figs. 4a and b, and S7). This reduction is significantly lower than that in North America, which required a 0.1% fuel sulfur limit in designated coastal regions [29,30]. Across different coastal regions, the SC region—most affected by shipping emissions—shows the greatest air quality improvement, with the adoption of ultra-low sulfur fuel reducing PM_2.5_ concentrations by more than 0.26 ​μg/m^3^. In the SEC region, due to the high density of inland ports in Jiangsu Province and the fact that major sea routes from the Bohai Bay to Shanghai are located farther from the mainland, significant concentration benefits are only observed under the S2 scenario, where the main shipping lanes fall within the regulated area. In the CBS region, due to the semi-enclosed nature of its sea area, the difference in control range between the S1 and S2 scenarios is relatively small, resulting in nearly the same concentration benefits. Consequently, there are no significant differences in health benefits between these two scenarios, with 448 and 508 cases of premature mortality attributed to long-term PM_2.5_ exposure avoided, respectively, across all coastal areas (Figs. 4f and g, and S8).

In the NECA scenarios, shipping NO_*x*_ emissions could be reduced by 35%, 36%, and 60% for N1, N2, and N3, respectively (the spatial characteristics of emission changes are shown in Fig. S9). Compared to SECA scenarios, the air quality benefits of NECA are more significant. In the N1 scenario, the annual average concentration of shipping-related PM_2.5_ across all coastal areas decreases by 0.12 ​μg/m^3^ to 2.16 ​μg/m^3^. However, the air quality impact from shipping remains relatively high compared to European regions where NECAs have been implemented [12]. In particular, in the central coastal region of Guangdong Province and the northern coast of the Bohai Rim, the shipping-related PM_2.5_ decreases by more than 1.00 ​μg/m^3^. However, the air quality benefit remains insignificant in the SEC region, similar to that in S1 (Figs. 4c and S6). In the N2 scenario, this concentration continues to decrease by 0.01 ​μg/m^3^, which is more significant in the SEC region, especially for the Shanghai-Ningbo-Zhoushan area, where the main shipping routes are farther from mainland areas (Fig. 4d). Compared to the emission-reduction benefits, the PM_2.5_ concentration benefits in the N3 scenario are also imbalanced. The shipping-related PM_2.5_ only further decreases by 0.01 ​μg/m^3^ on average (Fig. 4e). In the SC region, shipping-related PM_2.5_ can further decrease by up to 0.50 ​μg/m^3^. Additionally, since the 200-Nm control range extends far beyond the 12-Nm or port-only control zones, coastal cities on the Shandong Peninsula within the CBS region also observe PM_2.5_ reductions of approximately 0.10 ​μg/m^3^. In terms of health benefits, the N1, N2, and N3 scenarios avoid 1842, 2236, and 2639 cases of premature mortality, respectively, across all coastal areas (Figs. 4h–j, and S8).

**Fig. 5:**
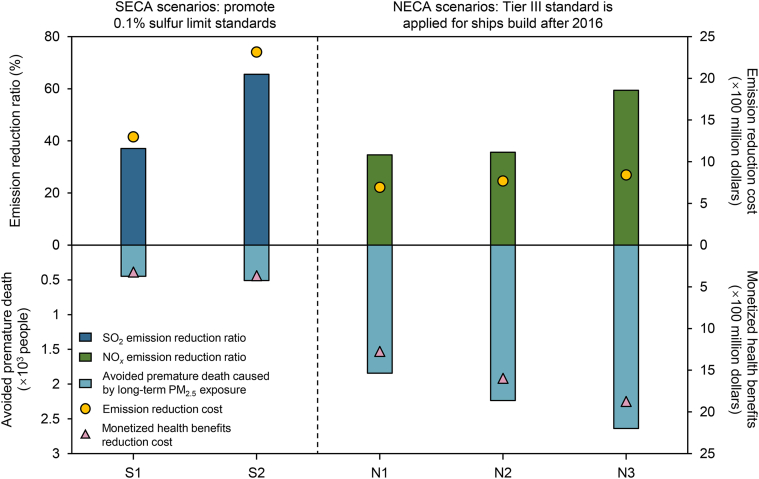
Cost-benefit analysis of different shipping emission control scenarios.

As shown in Fig. 5, the cost of SECA scenarios in terms of emission reduction and the benefits of avoided premature mortality are highly imbalanced. In the S1 scenario, the monetized health benefits can reach USD 322 million, while the fuel price premium amounts to USD 1.30 billion. In the S2 scenario, the total cost exceeds USD 2 billion. However, as previously noted, there is no significant improvement in population exposure compared to the S1 scenario, leading to only a slight increase in monetized health benefits of USD 42 million. The emission-reduction cost of implementing the Tier III standard, which combines pretreatment and post-exhaust-treatment NO_*x*_ reduction technologies, is USD 47 per kW. Therefore, the average cost of the NECA scenarios is USD 770 million. For the N1-N3 scenarios, the health benefits increase from USD 1.28 billion to USD 1.87 billion as the NECA coverage expands. This indicates that under the current level of shipping emissions in the seas around China, gradually reducing shipping NO_*x*_ emissions will lead to even greater benefits.

## 4 Conclusion

At China's current levels of shipping emissions, further promoting the use of ultra-low sulfur fuel for ships can achieve significant emission reductions, but provides limited air quality benefits except for certain coastal cities. Considering the high cost of ultra-low sulfur fuel, the overall benefits of this policy are further diminished. Therefore, unlike the continuously expanding SECA policy implemented between 2016 and 2019, the ultra-low sulfur fuel policy introduced in Hainan in 2022 is better suited for targeted implementation in densely populated port-city waters, such as Shanghai and Shenzhen, in order to achieve more substantial health benefits.

Although the formation of PM_2.5_ is influenced by atmospheric oxidizing capacity and often exhibits a nonlinear relationship with NO_*x*_ and VOC emissions, individually controlling shipping NO_*x*_ and VOC emissions can lead to an almost linear reduction in PM_2.5_ concentrations without causing an air quality penalty, as their absolute emission levels remain relatively low—except in cases of extremely large NO_*x*_ reductions, which present technical challenges. Compared to VOC control, NO_*x*_ emission reduction is more effective in mitigating PM_2.5_ pollution, which is further supported by the NECA policy scenario simulations (N1-N3). As a result, China should prioritize learning from the NECA implementation experiences of Europe and North America, beginning by piloting NECA in selected major ports to preliminarily evaluate its real-world environmental benefits, following its successful phased expansion of SECA.

Reductions in land-based NO_*x*_ and VOC emissions can significantly influence atmospheric oxidizing capacity and affect the formation of shipping-related PM_2.5_. In the nonlinear relationship between land-based emissions and PM_2.5_, shipping emissions constitute a relatively small portion of total anthropogenic emissions. Therefore, as China continues to advance land-based emission control, efforts to regulate shipping emissions—particularly through the NECA policy targeting NO_*x*_—should be coordinated with land-based mitigation strategies. Based on China's previous experience, emission controls for land-based sectors are typically implemented ahead of the maritime sector. Thus, it is essential for the maritime sector to collaborate with other relevant departments and set effective targets for shipping-based NO_*x*_ reductions. This can help avoid scenarios in which emissions are reduced but air quality fails to improve, as exemplified in Fig. 3c, where controlling shipping NO_*x*_ emissions under conditions of high land-based VOC reductions yields minimal air quality benefits. Moreover, due to the significant regional heterogeneity in the nonlinear relationship between shipping-related PM_2.5_ and land-based emissions, shipping emissions control strategies should be region-specific and targeted.

## CRediT authorship contribution statement

**Zhenyu Luo:** Writing – original draft, Methodology, Investigation. **Zhaofeng Lv:** Conceptualization. **Tingkun He:** Validation, Data curation. **Wen Yi:** Validation, Data curation. **Yongyue Wang:** Investigation, Formal analysis. **Kebin He:** Supervision, Conceptualization. **Huan Liu:** Supervision, Conceptualization.

## Data and code availability

Data and codes used during the current study are available from the corresponding author upon reasonable request.

## Declaration of competing interests

The authors declare no competing interests.
