# Supplementary material for: Data-driven assessment of air quality and health benefits from future shipping emission controls in coastal China
Source: Eco Environ Health. 2025 Nov 26;4(4):100203. doi: 10.1016/j.eehl.2025.100203 (PMC12723036; doi:10.1016/j.eehl.2025.100203)
Supplement: Multimedia component 1 [file mmc1.docx]

**Supplementary Materials**

**Data-driven assessment of air quality and health benefits from future shipping emission controls in coastal China**

Zhenyu Luo, Zhaofeng Lv, Tingkun He, Wen Yi, Yongyue Wang, Kebin He, Huan Liu^*^

^*^Correspondence to Huan Liu. E-mail: [liu_env@tsinghua.edu.cn](mailto:liu_env@tsinghua.edu.cn)

# Discriminator Model Description

In DeepShip, the dimensions of the predictors (F) are 136 × 137 × 14. The generator (G) of DeepShip model starts with a 3 × 3 convolutional layer that transforms the F with 14 input channels into 32 channels. This is followed by a series of eight residual blocks, each composed of two 3 × 3 convolutional layers and two normalization layers. In the convolutional layers of these blocks, the number of channels in the feature tensor remains at 32 to enhance the feature representation capabilities of the generator network. Subsequently, another 3 × 3 convolutional layer reduces the number of feature channels to 4, resulting in a tensor dimension of 136 × 137 × 4, which corresponds to the spatial distribution of the four main components of shipping-related PM_2.5_ (sulfate, nitrate, secondary organic matter, and primary organic matter). After the final 3 × 3 convolutional layer, the output is the spatial distribution of shipping-related PM_2.5_, with dimensions of 136 × 137 × 1. Additionally, following the eight residual modules, there is a branch with the same architecture as the model for generating the shipping-related PM_2.5_ and its component concentrations. This branch outputs the spatial distribution of each component concentration in the total PM_2.5_, as well as the total PM_2.5_ concentration, to support multi-task learning.

The goal of generator G is to predict the shipping-related PM_2.5_, while discriminator D is to differentiate between the shipping-related PM_2.5_ generated by G and that simulated by CMAQ, both with dimensions of 136 × 137 × 1. After processing the input samples through a 4 × 4 convolutional layer, the feature channels increase to 64. Subsequently, the feature tensor is fed into a structure consisting of three convolutional layers and normalization layers, with the number of feature channels increasing from 128 to 256. The feature tensor then enters a residual module, which also comprises three convolutional layers and normalization layers, with feature channels of 128, 128, and 256, respectively. Finally, after passing through a fully connected layer, the feature tensor is converted into an output, where a value of 1 denotes a positive classification and 0 indicates a negative classification.

# Shipping Emission Inventory Model

The Shipping Emission Inventory Model (SEIM v2.0) ^4^ is a disaggregate dynamic method driven by driven (a) the high-frequency ship Automatic Identification System (AIS) data, including signal time, coordinate location, navigational speed, and operating status, and (b) the integrated Ship Technical Specifications Database (STSD) (updated to 2020), which describes ship static properties, including vessel type, maximum designed speed, DWT and engine power. First, the originally collected raw AIS data and ship profile data from multiple sources are combined to form a ship activity database and STSD, and the RVs are identified based on the ship trajectories. Second, a route restoration module is applied for cross-land trajectory with a long distance in the AIS data, in which the 10 min linear interpolation will be applied on the shorted paths instead. Third, the instantaneous emission along with the movement of the ship’s trajectory will be calculated based on the ship’s static technical parameters, dynamic load changes, and extra parameters and factors. Then, the policy-compliant modification will be applied for vessels entering the DECAs to switch to low-sulphur fuels (LSFs). Finally, shipping emission inventory datasets will be established and used for visualization and analyses from multiple perspectives. In the SEIM, shipping emissions for both air pollutants (e.g., SO_2_, PM, NO_x_, CO and HC) and greenhouse gases (e.g., CO_2_, CH_4_ and N_2_O) from the main engines, auxiliary engines and boilers were calculated.

$$E^{\mathrm{ME}}=\sum_{t=1}^{n} MCR\times\mathrm{EF}_{p,i,j,l}^{\mathrm{ME}}\times\mathrm{LF}_{t}\times\mathrm{LLAF}_{p}\times{{\Delta T}_{t}\times10}^{-6}$$

$$E^{\mathrm{AE}}=\sum_{t=1}^{n} P_{v,s,m}^{\mathrm{AE}}\times\mathrm{EF}_{p,i,k}^{\mathrm{AE}}\times{{\Delta T}_{t}\times10}^{-6}$$

$$E^{\mathrm{Boiler}}=\sum_{t=1}^{n} P_{v,s,m}^{\mathrm{Boiler}}\times\mathrm{EF}_{i.p}^{\mathrm{Boiler}}\times{{\Delta T}_{t}\times10}^{-6}$$

Where $\mathrm{MCR}$ is the maximum continuous rated power (kW) for each vessel; $\mathrm{EF}_{p,i,j,l}^{\mathrm{ME}}$ is the emission factor for fuel type *i*, engine type *j*, emission standard k and species p (g/kW·h); $\mathrm{LF}_{t}$ is the load factor in time interval *t*, $\mathrm{LLAF}_{p}$ is the low load adjust factor for species *p*, which is applied when the load factor is less than 20%; ${\Delta T}_{t}$ is the time interval of the *t*-th continuous AIS signal (h); *n* is the total number of AIS signal time intervals under each category. $P_{v,s,m}^{\mathrm{AE}}$ and $P_{v,s,m}^{\mathrm{Boiler}}$ is the operating power (kw) of AEs and boiler of ship type *v* and size bin *s* (divided by dead weight tonnage) under operating mode *m* (kW); $\mathrm{EF}_{p,i,k}^{\mathrm{AE}}$ is the emission factor of pollutant *p* for AEs using fuel type *i* and complying with emission standard *k* (g/kW·h); $\mathrm{EF}_{i.p}^{\mathrm{Boiler}}$ is the emission factor of pollutant p for boilers using fuel type *i* (g/kW·h).

# Mortality estimation

Here we calculated the resulting health endpoints from PM_2.5_ exposures by chronic obstructive pulmonary disease (COPD), ischemic heart disease (IHD), stroke, and lung cancer ^17^. We used the integrated risk function developed by Burnett et.al.^17^ to quantify the health impacts. The equations are shown as follows:

$$RR\left( C \right)=\left\{ \begin{aligned} 1+\alpha\left( 1-e^{-\gamma(C-C_{0})^{\theta}} \right), if C< C_{0} \\ 1, else \end{aligned} \right.$$

$$AF= \frac{RR-1}{RR}$$

$$E=AF\times M\times P$$

where $C$ is the annual PM_2.5_ concentrations, $C_{0}$ is the concentration threshold over which there will be health damages, and $\alpha$, $\gamma$, and $\theta$ are parameters that describe the shape of the CRF curve (Table S6). After we obtained the relative risks caused by the exposure $RR$, we calculated the attributable fraction $AF$ which indicates the proportion of incidents in the population that are attributable to the PM_2.5_ concentrations. And finally, we multiplied the attributable fraction $AF$ for each aging group by the cause-specific cross-sectional mortality rate $M$ and the population $P$ at each age group, and we got the excess mortalities caused by PM_2.5_ concentrations in each age group $E$.

Here, we used yearly (2016-2020) city-level population data and provincial age-structure data from Chen’s study ^20^. The yearly age-structured cause-specific cross-sectional mortality rate for COPD, IHD, stroke, and LC was from Chinese disease surveillance system death surveillance data set.

# Figures


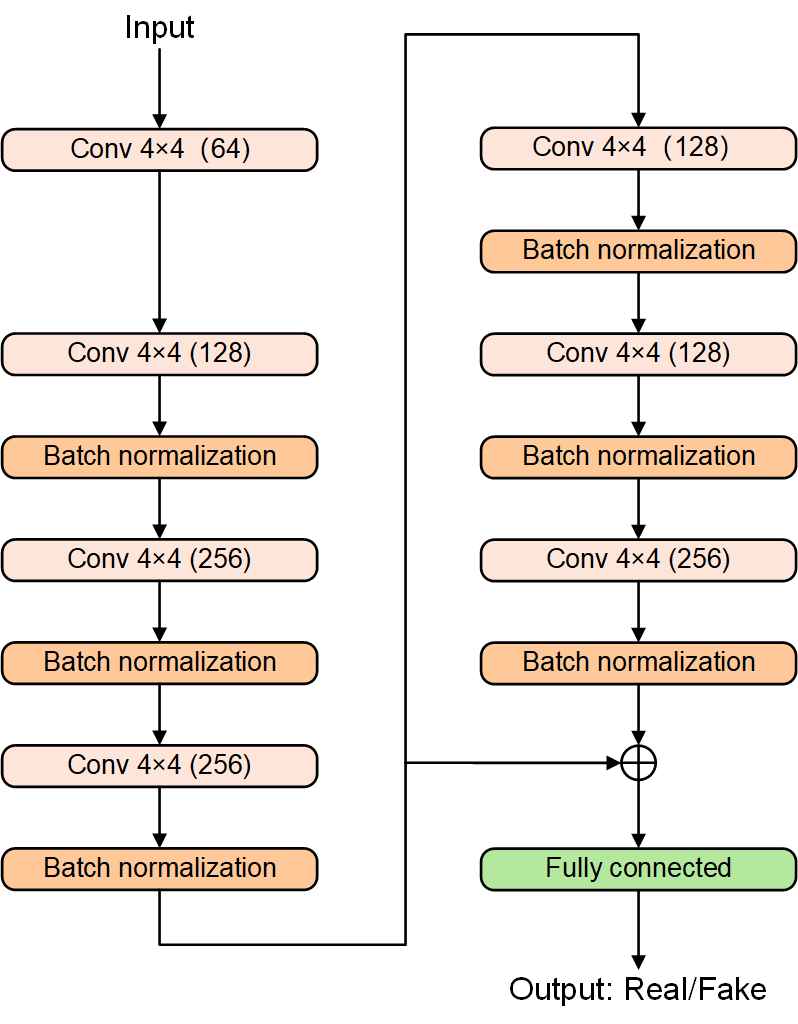


Figure S1 The structure of the discriminator.


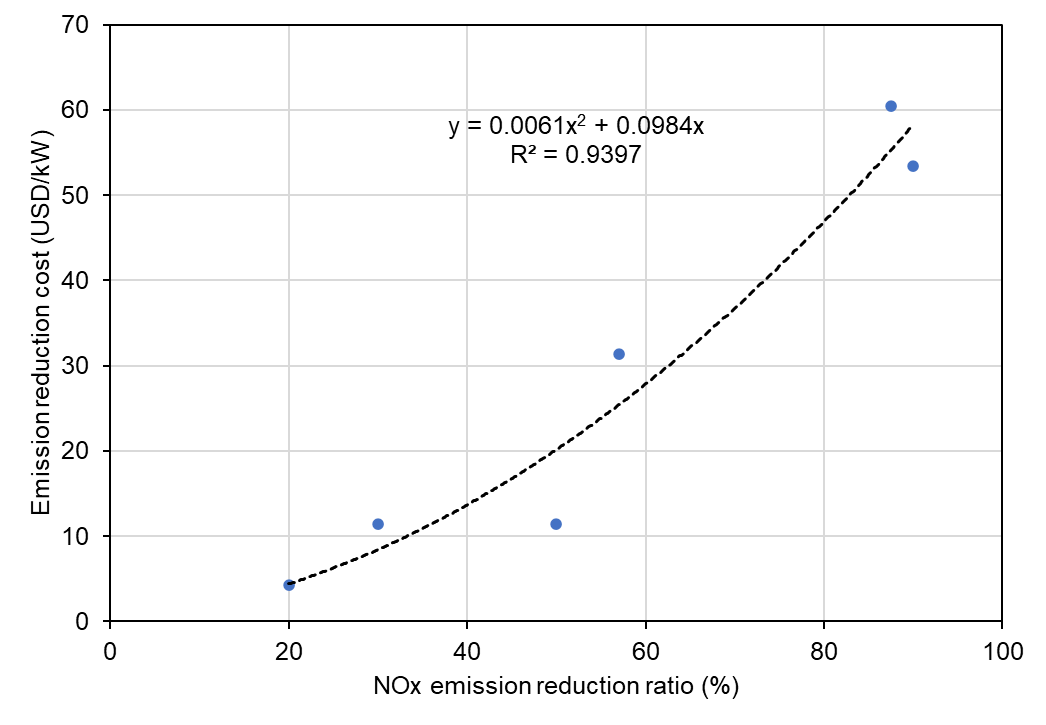


Figure S2. The fitting relationship between NOx emission reduction ratio and cost.


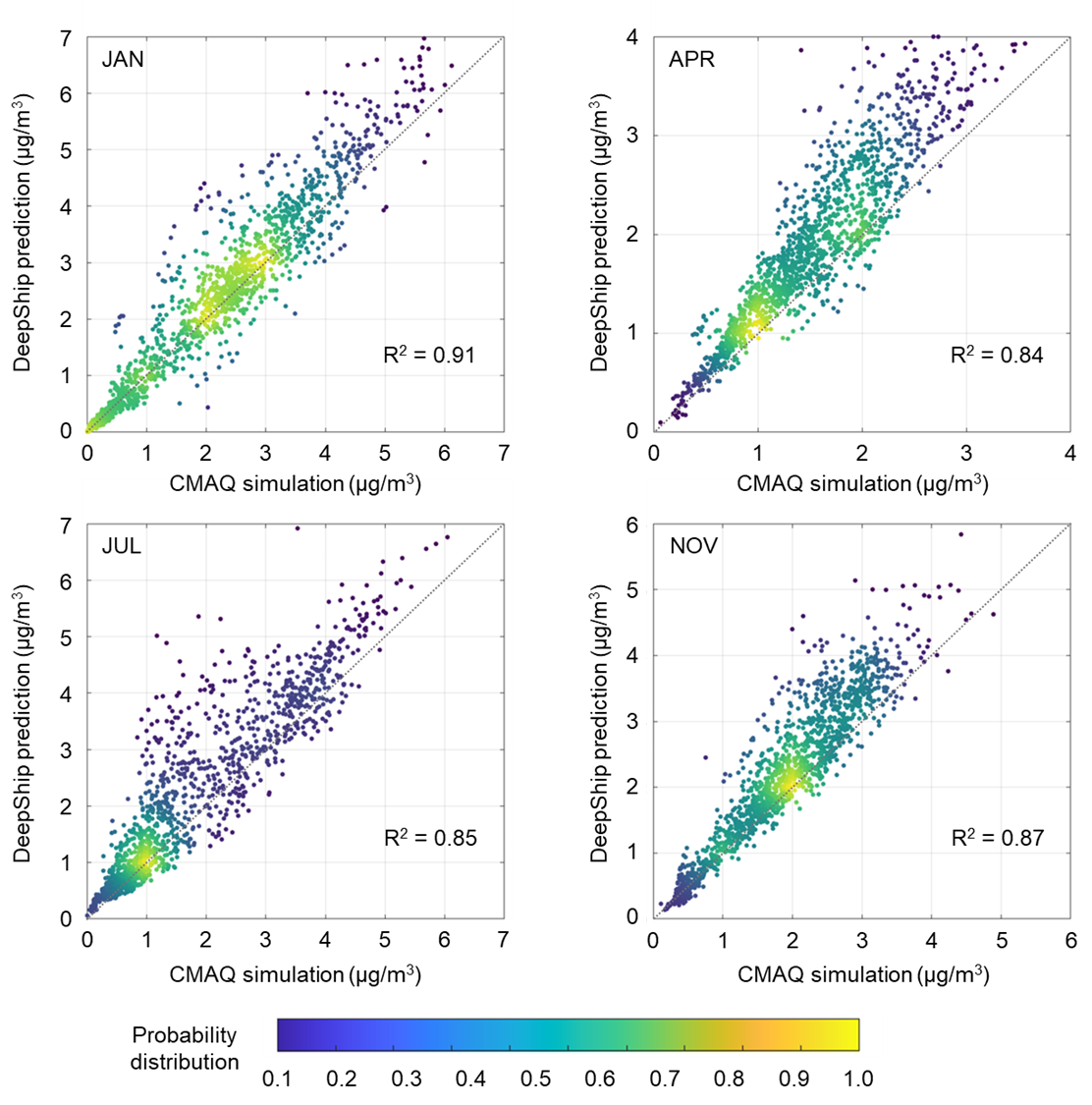


Figure S3. Comparison of shipping-related PM_2.5_ from DeepShip and CMAQ.


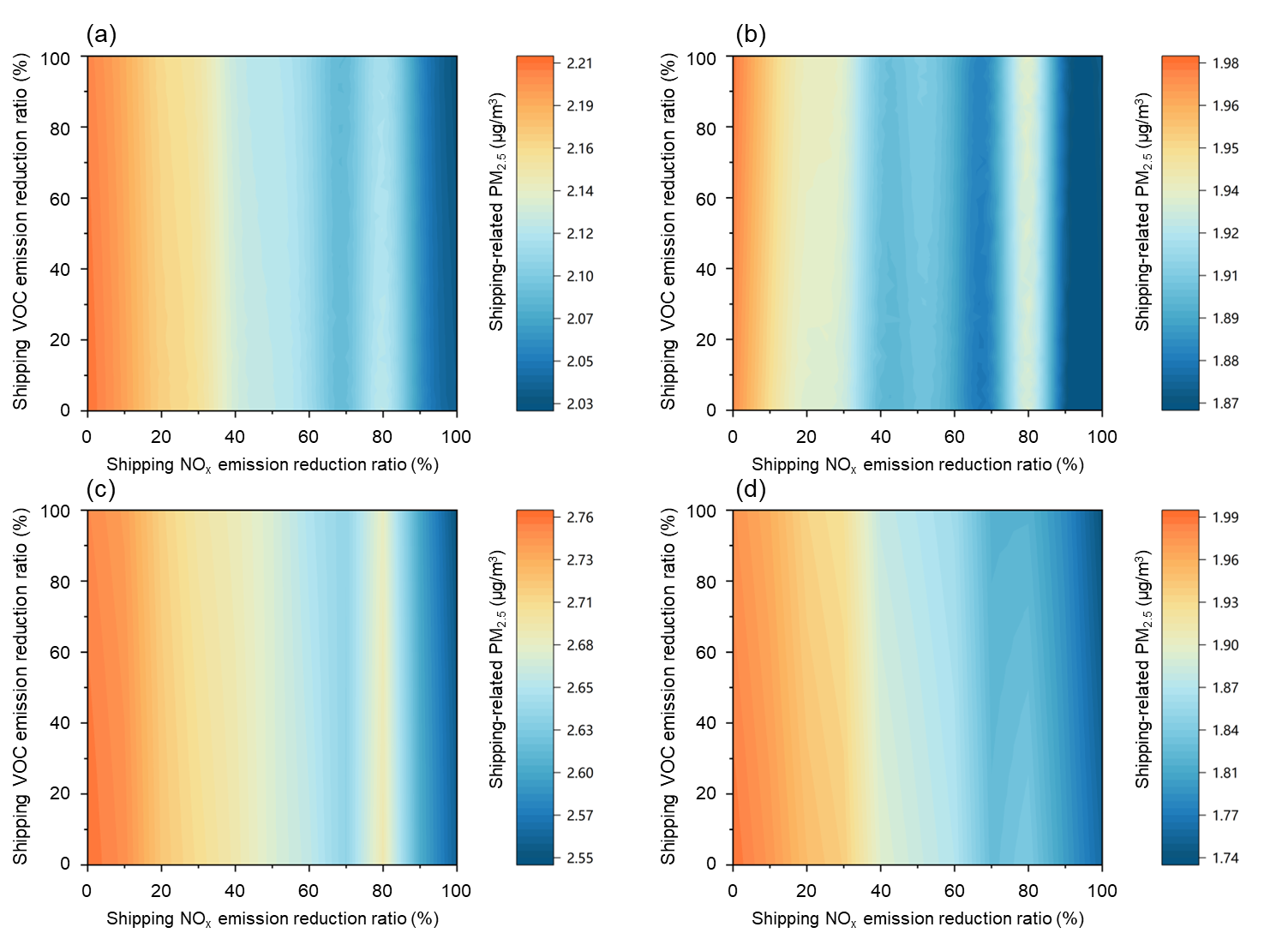


Figure S4. The response of shipping-related PM_2.5_ to changes in shipping NO*_x_* and VOC emissions for (a) all coastal areas, (b) CBS region, (c) SEC region and (d) SC region.

Note. Different from Figure 3, these figures show the shipping-related PM_2.5_ concentration values rather than the changes in concentration.


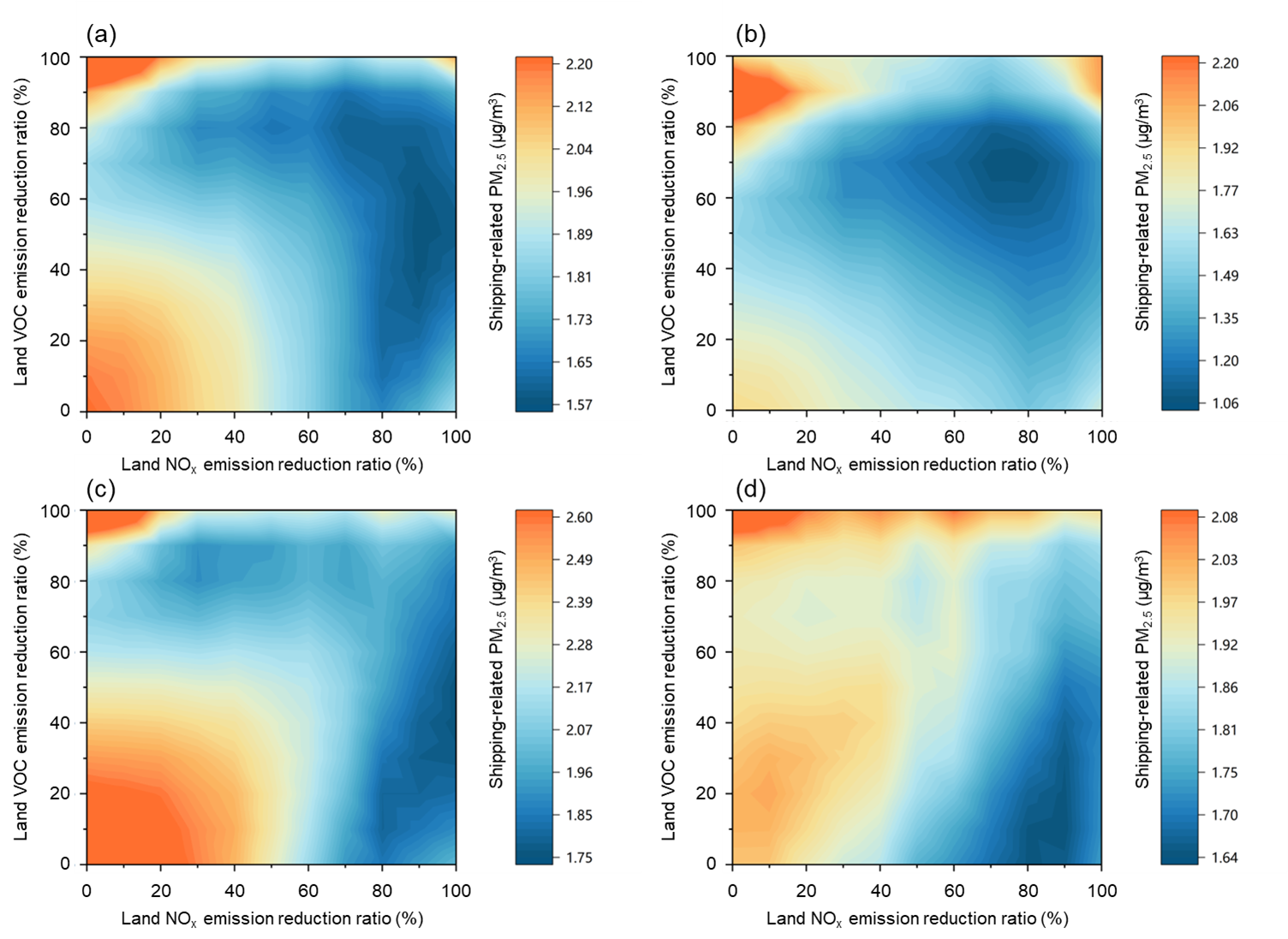


Figure S5. The response of shipping-related PM_2.5_ to changes in land-based NO*_x_* and VOC emissions for (a) all coastal areas, (b) CBS region, (c) SEC region and (d) SC region.

Note. Different from Figure 3, these figures show the shipping-related PM_2.5_ concentration values rather than the changes in concentration.


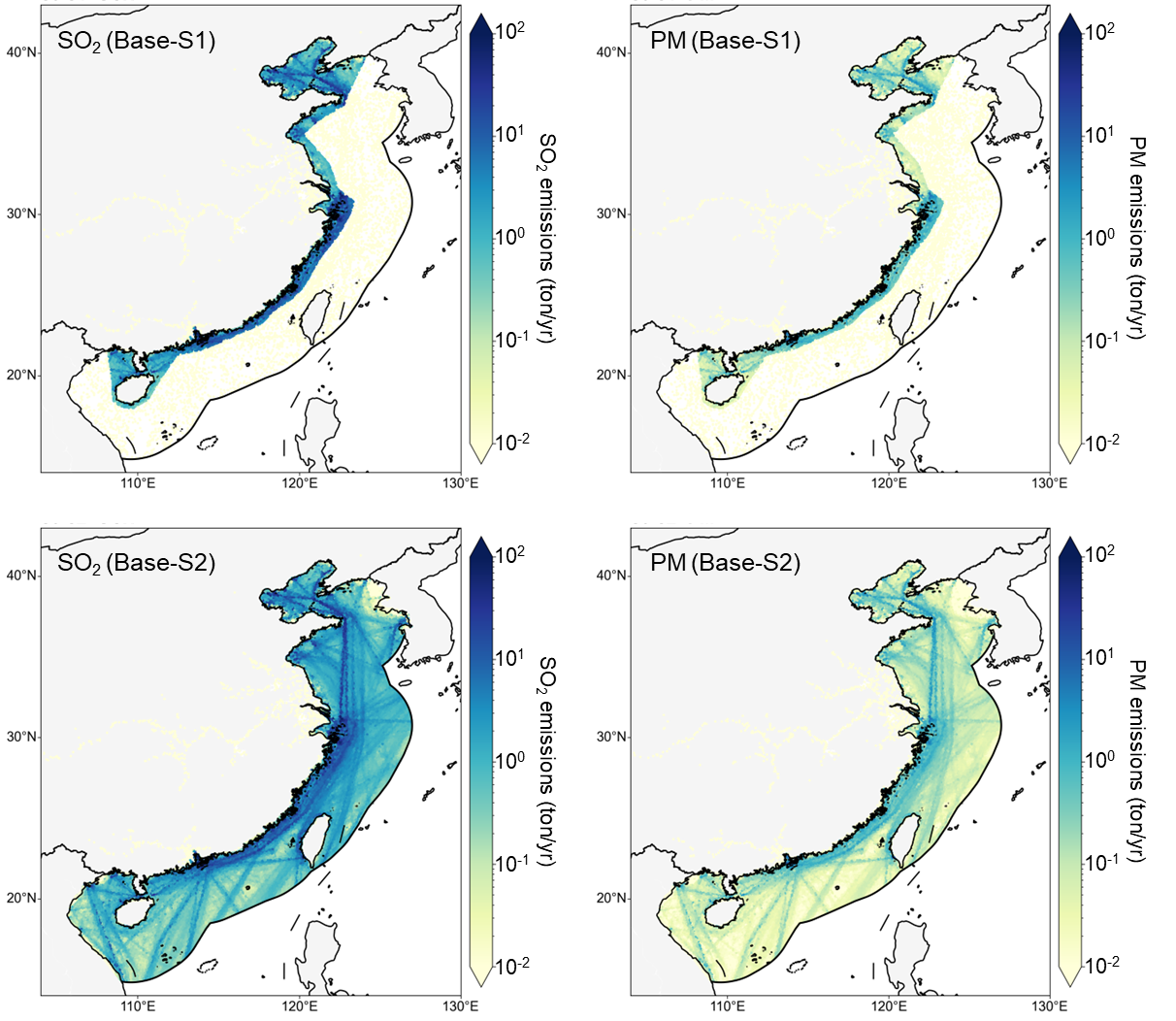


Figure S6. Shipping emissions changes for SO_2_ and PM in S1 and S2 scenarios.


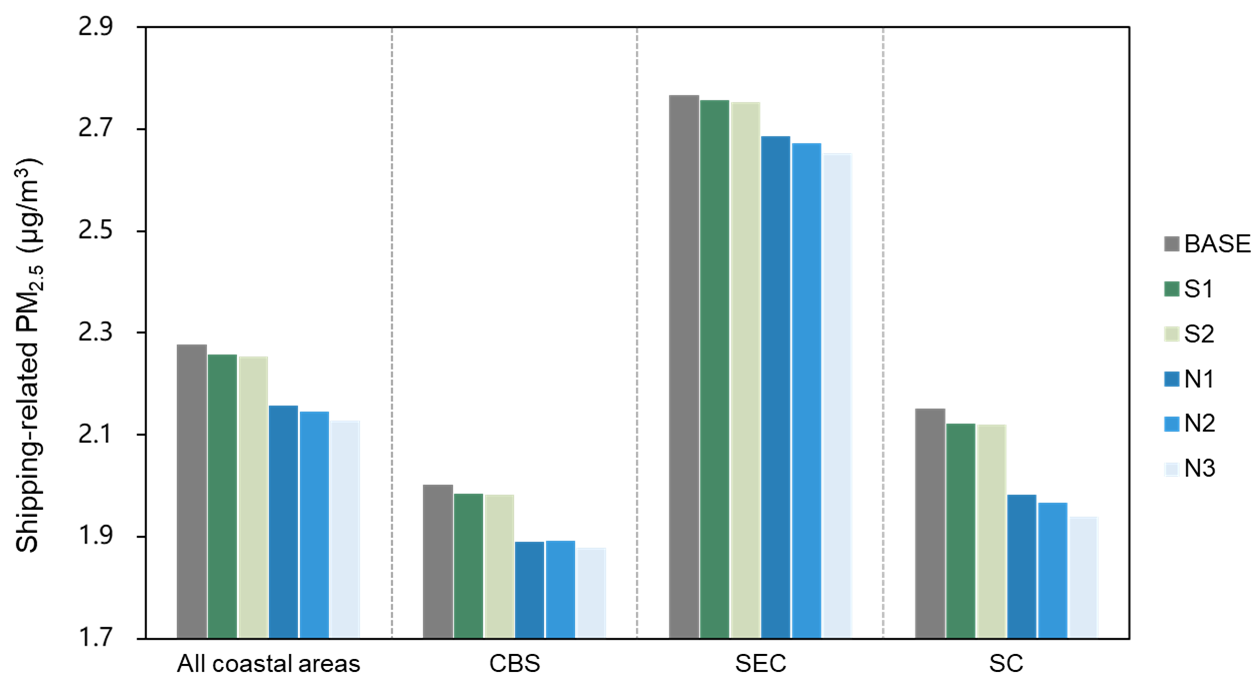


Figure S7. Shipping-related PM_2.5_ for different scenarios.


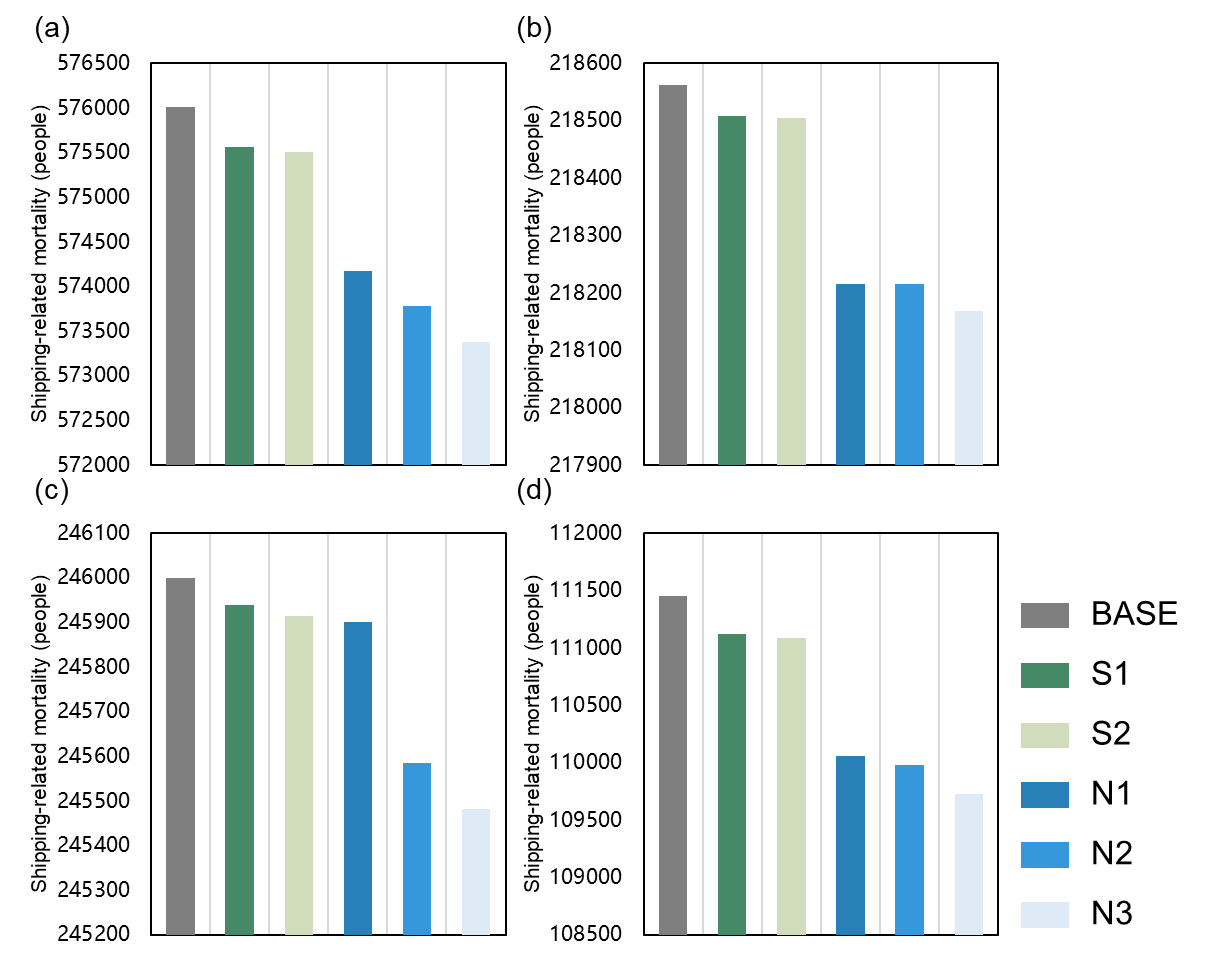


Figure S8. Shipping-related mortality for different scenarios for (a) all coastal areas, (b) CBS, (c) SEC, and (d) SC.


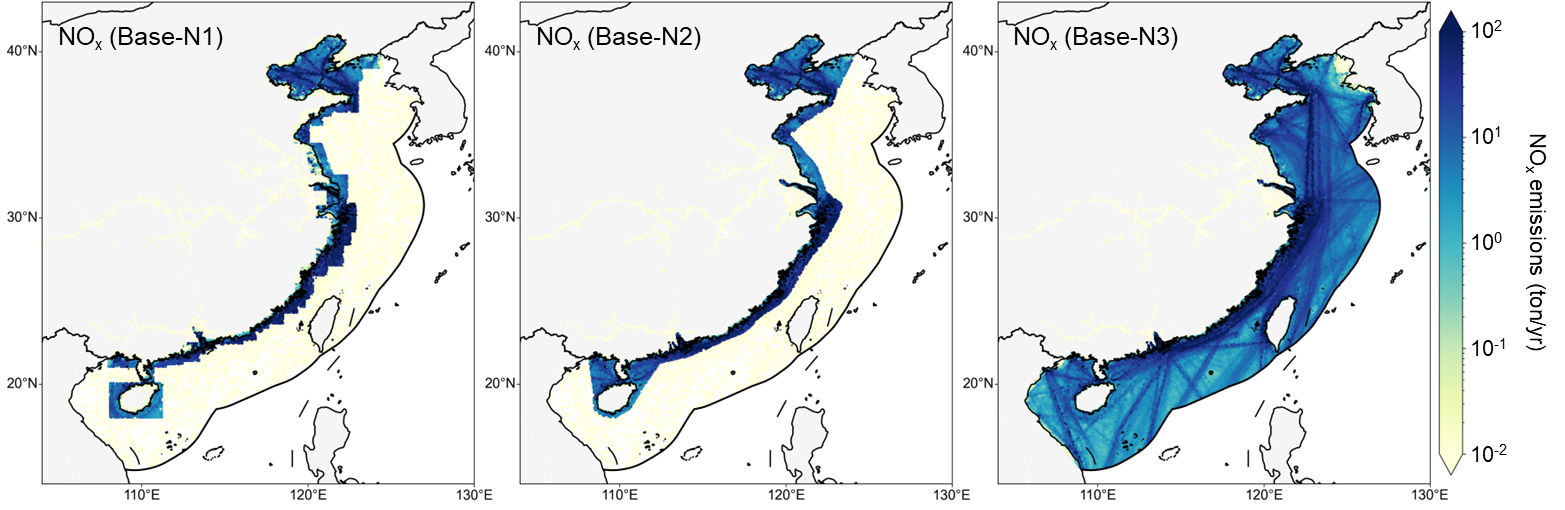


Figure S9. Shipping emissions changes for NO*_x_* in N1, N2 and N3 scenarios.

# Tables

Table S1. Emissions used in the CMAQ model.

| Emissions | Base Year | Reference |
| --- | --- | --- |
| Shipping Emissions | 2016-2019 | SEIM ^4^ |
|  | 2020 | SEIM ^6^ |
| Land-based anthropogenic emissions in China (mobiles, industry, power, domestic, and agriculture) | 2016-2020 | MEIC  (<http://www.meicmodel.org/>,  last access: September, 2025) |
| Anthropogenic emission from other countries within the modeling domain | 2010 | MIX emission inventory ^30^ |
| Open burning | 2015 | Cai et al ^31^ |
| Biogenic emissions | Real | Model of Emissions of Gases and Aerosols from Nature (MEGANv3, <https://sites.google.com/uci.edu/bai>, last access: September, 2025) |
| Emissions from windblown dust | Real | In-line calculated in CMAQ  (https://www.epa.gov/cmaq, last access: September, 2025) |
| Emissions from sea salt | Real | In-line calculated in CMAQ  (https://www.epa.gov/cmaq, last access: September, 2025) |

Table S2 Description of virtual emission reduction scenarios.

| Emission source | Pollutants | Reduction ratio (%) | Count |
| --- | --- | --- | --- |
| Shipping | SO_2_ | 10, 20, 30, … ,100 | 10 |
| Shipping | NO*_x_*–VOC | 10-10, 10-20, 10-30, …, 10-100  20-10, 20-20, 20-30, …, 20-100  30-10, 30-20, 30-30, …, 30-100  …  100-10, 100-20, 100-30, …, 100-100 | 100 |
| Land-based anthropogenic source | NO*_x_*–VOC | 10-10, 10-20, 10-30, …, 10-100  20-10, 20-20, 20-30, …, 20-100  30-10, 30-20, 30-30, …, 30-100  …  100-10, 100-20, 100-30, …, 100-100 | 100 |

*For the NO*_x_* and VOC co-reduction scenarios, the two numbers in the “Reduction ratio (%)” column represent the reduction percentages of NO*_x_* and VOC, respectively. For example, 10–20 indicates a 10% reduction in NO*_x_* and a 20% reduction in VOC.

Table S3 Description of potential policy scenarios.

| Scenarios | Description |
| --- | --- |
| Base | 2020 emission level |
| S1 | Implementation of ultra-low sulfur fuel with a sulfur limit of 0.1% m/m within 12 Nm of the Chinese mainland territorial baseline |
| S2 | Implementation of ultra-low sulfur fuel with a sulfur limit of 0.1% m/m within 200 Nm of the Chinese mainland territorial baseline |
| N1 | Ships built after 2016 must comply with Tier III standards, applicable to all port waters in China |
| N2 | Ships built after 2016 must comply with Tier III standards, applicable within 12 nautical miles of the Chinese mainland territorial baseline |
| N3 | Ships built after 2016 must comply with Tier III standards, applicable within 200 nautical miles of the Chinese mainland territorial baseline |

Table S4 NO*_x_* emission reduction potential and costs of different technologies

| Technology | | | Reduction ratio(%) | Cost (USD/kW) | |
| --- | --- | --- | --- | --- | --- |
|  |  |  |  | 中速机 | 低速机 |
| Fuel optimization | Fuel emulsification | | 20–80 | 13.4 | 9.5 |
|  | Fuel additives | | 13–45 | - | - |
|  | Fuel substitution | Methanol | 60 | 5.2 | 3.4 |
|  |  | Liquefied natural gas | 86 |  |  |
| Combustion pre-treatment technologies | Two-stage turbocharging | | - | - | - |
|  | Miller cycle | | 20–60 | - | - |
|  | Fuel injection systems | | 15–42 | - | - |
|  | Water-based strategies | Humidification technology | 40–65 | - | - |
|  |  | Water injection technology | 42–70 | 32.4 | 30.3 |
|  | Dual-fuel engine technology | | 40–80 | - | - |
| Exhaust gas after-treatment technologies | Exhaust gas recirculation | | 80–85 | 13.2 | 9.6 |
|  | Selective catalytic reduction (SCR) | | 80–95 | 60.8 | 60.1 |
|  | Low-temperature plasma technology | | 60 | - | - |
|  | Seawater scrubbing (desulfurization) technology | | - | 69.8 | 55.2 |
| Combined technologies | SCR + seawater scrubbing | | 90 | 44.5 | 53.4 |
|  | SCR + light diesel | | 90 | 48.6 | 58.3 |

Table S5 Total ship power for N1, N2, and N3 scenarios.

|  | N1 | N2 | N3 |
| --- | --- | --- | --- |
| Total ship power (kW) | 14563736 | 16181929 | 17696554 |

Table S6. The shape parameters of CRF of chronical health endpoints ^40^.

| Health Endpoints | **α** | **γ** | **δ** | **C_0_** |
| --- | --- | --- | --- | --- |
| IHD | 0.83 | 0.0717 | 0.5516 | 6.96 |
| Stroke | 1.01 | 0.0174 | 1.1244 | 8.38 |
| COPD | 29 | 0.0005938 | 0.6786 | 7.17 |
| LC | 33.49 | 0.00005013 | 1.0128 | 7.24 |
